# Case of Poisoning by Alcoholic Extract of Belladonna

**Published:** 1868-02

**Authors:** H. A. Dubois

**Affiliations:** Assistant-Surgeon U.S.A.


### CASE OF POISONING BY ALCOHOLIC EXTRACT OF BELLADONNA.

As the following case presents several points of interest, I transmit it with but few comments:

December 28, 1866, feeling indisposed, I ordered for myself: Sulph. quin., grs. v.; ext. coloc. do., grs. x.; pil. mass. hyd., v., M. Ft. pil. No. iv.

I received the four pills, two of which I at once took, and soon after retired (about eleven A.M.) I slept but little, but was not very restive, and did not observe anything peculiar in my sensations until near eight A.M. the following morning when on putting my foot on the floor, I came near loosing my balance and staggared some distance across the room, unable to support myself steadily, or regulate the movements of my legs. Dressing with much difficulty, I managed to reach the surgery, where, seating myself, I commenced prescribing for the sick. I now, for the first time, found that I was unable to read the names on the sick report, the page appearing perfectly blank. Up to this time I had not been able to control my thoughts. On rising, I remember that I had a faint idea that something was wrong, but was unable to say what; the acts in putting on my clothes were performed nearly involuntarily, the prominent idea being the necessity I was under of being punctually at my post at sick call. Not seeing any names on the sick report, I asked the steward the reason, and then discovered that I could not control the movements of my tongue. I managed, however, to make myself understood, and was informed that the names were there. I remember that then, by a strong impulse, I collected my thoughts to endeavor to ascertain the cause of my disability. The first thought which presented itself was that I was drunk, but this I rejected, as I could not remember having drunk any intoxicating liquor the preceding day. Next, I ran over my actions the evening before, and I recalled the two pills I had taken, recollected their composition with difficulty, and the thought then struck me that as the extract of colocynth and extract of belladonna were both put up in pots much alike, one had been mistaken for the other. I remember that I recalled the amount I had probably taken, and determined that I would prescribe for the sick, if I could neither see, speak distinctly, nor stand steadily, before I retired to my room. I ordered the steward to call the names out, and when the first man came up I could see his outline, but could not recognize his features. I, however, remembered his name and what should be the matter with him, and having his last prescription read out, I continued it, afraid to trust myself in prescribing. In this way, with few changes, and then careful to use the simplest of medicines, I saw, or rather did not see, some thirty sick, for each of whom I prescribed, or continued his former prescription.

I then returned to my room with a brain whirling, a dull, heavy pain in the back of the head, and my mind wandering from time to time. I recollect distinctly trying to collect my thoughts, and using the utmost exertions of my will to keep them fixed, so as to try to remember the smallest fatal dose of the extract of belladonna, and to recall all the symptoms of poisoning from that drug, and their proper treatment. I came to the conclusion that I would treat myself on general principles. I had some strong coffee brought and put before me, and drank cupful after cupful. I sent for whiskey, and mixing it with the coffee, I continued its use, taking also, during the day, several half grain pills of morphiæ sulph.

This treatment relieved the giddiness. I found my mind ceased to wander, and though a vague feeling—a feeling of vacuity—of a lack of something, still continued, together with a severe pain in the back of the head whenever I tried to think, I felt better. I now tried to examine my eyes in a glass, but could only see a general outline. However, by going across to the other side of the room and looking at the glass, I managed to discover that my pupils had expanded so that I could see no iris. As I now look back to this period, everything—my thoughts and actions—all seem vague and misty; though I remember them, and that distinctly, still the thoughts seem to have formed themselves under the power of a strong effort of the will, and that the least relaxed, the thought would escape. So too, with my actions. In walking, I can remember that on fixing one foot on the ground, it required an effort of the will to raise the other, and much judgment in so placing it that the body's centre of gravity was preserved. My arms and legs—but the latter not to the same extent—felt numb and cold to my touch. With great difficulty I held anything in my hands. By noon, or four hours since I first noticed the effects of the poison, I was able to visit and prescribe for some twenty patients in hospital, but returned greatly exhausted. This state continued all the afternoon and evening, and I passed a sleepless night; my mind wandering, and vague and misty visions presenting themselves. For two days my vision continued much impaired, and it gradually became normal as the pupils resumed their usual size.

While my vision was most impaired, I noticed objects nearly as well as ever a mile or two out on the prairie, though if I looked attentively, the effort became exceedingly painful. The action of the brain slowly became normal. Exertion of any kind—even the reading of the simplest book—caused intense fatigue, and a dull, heavy pain in the back of the head. These symptoms, together with great lassitude, continued for two or three weeks. There are a few symptoms that I have neglected to mention. Colicky pains and dryness of the mouth and throat presented themselves on the second day. The dejections for several days were dark colored, and extremely offensive, and the urine was voided frequently and in large quantities. Some days after these symptoms, I noticed on my hand a large red blotch, which disappeared on pressure, and which, in the course of a week, faded, leaving the skin rough. For two months I felt lassitude, and but little mental exertion was required to bring back the peculiar feeling in the head.

I have described this case with minuteness, as its chief point of interest seems to me to reside in the control the will appears to have had over the cerebrum, constantly bringing the mind under control, and holding it to its work; and in the remembrance of things, and their appearance, seen with a brain and through optic nerves poisoned with belladonna.

In conclusion, I will only add that the amount of poison taken was five grains. The preparation used was Squibb's alc. ext., and, as I had supposed, it had been used for the ext. coloc. co. by the soldier whom I had been compelled to use as a steward.—H. A. Dubois, M.D., Assistant-Surgeon U.S.A., in the *Medical Record.*

